# Anatomy—Descriptive and Surgical

**Published:** 1887-12

**Authors:** 


**Anatomy—Descriptive and Surgical.** By HENRY GRAY, F. R. S., Lecturer on Anatomy at St. George's Hospital Medical College. Edited by T. Pickering Pick, Surgeon to, and Lecturer on Surgery at St. George's Hospital. A new American, from the eleventh English edition, thoroughly revised and re-edited, with additions by William W. Keen, M. D., Professor of Surgery in the Women's Medical College of Pennsylvania, to which is added "Landmarks—Medical and Surgical." By LUTHER HOLDEN, F. R. C. S., with additions by William W. Keen, M. D. Philadelphia: Lea Brothers & Co. 1887.

"Gray's Anatomy" is the one book which the medical student or practitioner cannot do without, and the appearance of a new edition is an event of importance. About five years ago, a new edition appeared, which, at that time, seemed to be as nearly perfect as possible; but the present book shows many valuable improvements upon the tenth edition. The American editor thus summarizes the chief changes: "In the section on the brain, I rejected the English cuts showing the sulci and convolutions, and substituted the more accurate and generally-adopted plates of Ecker. The text was correspondingly inaccurate, and could not be adapted to the new illustrations without a few changes, which, it is hoped, will facilitate the study of this difficult part of anatomy. I have also carefully described the cerebral circulation, and have added a section on cerebral localization and topography—subjects of great and increasing importance, especially in view of the recent rapid strides in cerebral surgery. The chief anatomical text-book in the English language ought not, I think, to be behind in this department, but should lend its aid to further progress by giving the fullest and most accurate knowledge attainable. Among the other additions to the text may be noted also paragraphs on the action of the muscles of the foot-sole; a more accurate description of the pectoralis major, supinator longus, and thenar muscles; and a careful description of the palmar fascia. In all, 113 new engravings have been added, of which many are original. These, with their descriptive matter, and likewise all my other additions, have been distinguished by brackets. Among these new illustrations are many of the most obvious utility, such as a series of sections through important joints; a series of frozen sections through the trunk, the extremities, and the female pelvis; cuts illustrating the histology of various tissues; the shoulder and pelvic girdles; the interior of the nose and the larynx; the development and occlusion of the teeth, and the absorption of the alveolar processes; the structure of the muscles; the ligamentum nuchæ; the occipito-frontal and interosseous muscles; the palmar fascia; a series giving the points for the application of electricity to the muscles; a series on the circulation of the brain and spinal cord; another series to illustrate cerebral localization and topography; another, on the cutaneous distribution of the nerves; a number of cuts to elucidate the anatomy of the cerebrum; two showing the sympathetic nerve; and others illustrating the peritoneum, the muscularis mucosæ, the female perineum, and the genito-urinary organs of both sexes. Whereever practicable, colors have been introduced to distinguish the veins, arteries and nerves, so that, in the colored edition, the American additions shall be in harmony with this novel feature of the latest English original." There is scarcely a section of the work, therefore, which has not been extensively enriched in the matter of illustrations. Following the English edition, the arteries, nerves and veins in the wood-cuts have been colored. This adds decidedly to the beauty of the illustrations, if not to their value, and the additional expense being but slight, we think the colored edition will be popular with the students. The publishers have issued the book in very handsome form, and indeed, as a whole, it is very near perfection.

